# EBP50 induces apoptosis in macrophages by upregulating nitric oxide production to eliminate intracellular *Mycobacterium tuberculosis*

**DOI:** 10.1038/srep18961

**Published:** 2016-01-05

**Authors:** Yang Guo, Yating Deng, Zikun Huang, Qing Luo, Yiping Peng, Jie Chen, Hong Jiang, Jianqing Ye, Junming Li

**Affiliations:** 1 Department of Clinical Laboratory, The First Affiliated Hospital of Nanchang University, Nanchang, 330006, China; 2 Department of Tuberculosis, Jiangxi Chest Hospital, Nanchang, 330006, China

## Abstract

*Mycobacterium bovis* BCG is known to have the capacity to inhibit the positioning of iNOS on BCG-containing phagosomes by interfering with EBP50, a scaffolding protein that controls the recruitment of inducible nitric oxide synthase (iNOS) at the vicinity of phagosomes in macrophages. However, knockdown of the expression of EBP50 still facilitates the intracellular survival of BCG, which suggested that EBP50 may have some other unknown antimycobacterial properties. In this study we show that overexpression of EBP50 by a recombinant lentivirus had no effect on the iNOS recruitment to *M.tuberculosis*-containing phagosomes, but significantly promoted the elimination of intracellular *M.tuberculosis*. We revealed in the present study that the enhancement of intracellular killing to *M. tuberculosis* upon EBP50 overexpression was due to the increased level of apoptosis in macrophages. We showed that EBP50 overexpression significantly increased the expression of iNOS and generation of nitric oxide (NO), and EBP50-induced apoptosis was NO-dependent and mediated by Bax and caspase-3. We found that *M. tuberculosis* decreases while *Mycobacterium smegmatis* increases the expression of EBP50 in RAW264.7 cells, which suggested that virulent mycobacteria are capable of modulating the antimycobacterial properties of macrophages by inhibiting the expression and interfering with the function of EBP50.

*Mycobacterium tuberculosis*, the pathogen causing tuberculosis, is one of the most successful pathogens of mankind. It is estimated that approximately one-third of the world’s population is infected with *M. tuberculosis*. In most cases, *M. tuberculosis* establishes latent infection in humans. From an estimated 2 billion people that have been infected with *M. tuberculosis*, less than 10% may develop symptomatic tuberculosis, indicating that the host immune system constrains *M. tuberculosis* replication in the majority of infected individuals^1^,^2^. On the other hand, *M. tuberculosis* produces neither toxins nor evasive enzymes; thus, the pathological lesion of tuberculosis also results from the immune response of the host. Therefore, the immune response of the host critically influences the progression of *M. tuberculosis* infection, and understanding the interaction between *M. tuberculosis* and host is crucial for controlling tuberculosis^3^.

Macrophage-mediated innate immune response functions as the first line of host defense against *M. tuberculosis*^3^,^4^. Once inhaled into the lungs of the host, the first encounter of *M. tuberculosis* is with alveolar resident macrophages. As one of the important immune effector cells, macrophages can phagocytose and eliminate intracellular microbes by multiple bactericidal mechanisms, including acidification of the phagosomes, and delivering of phagosomes to the lysosomes for degradation, generating bactericidal free radicals, such as reactive oxygen and nitrogen species, activating programmed cell death. Activated macrophages produce and release proinflammatory cytokines and chemokines, attract and activate monocytes and other inflammatory cells to infection sites^5^,^6^,^7^,^8^. Thus macrophages are crucial for human antimycobacterial defense. However, macrophages have been identified as the main host cell of *M. tuberculosis* in humans^9^,^10^, which indicated that *M. tuberculosis* has evolved mechanisms to counter bactericidal effectors of macrophages. Unfortunately, although extensively studied, the mechanisms of *M. tuberculosis* to defend against macrophages have not yet been elucidated completely.

EBP50, also known as Na^+^/H^+^ exchange regulatory factor (NHERF1), is a PSD-95/Dlg-1, Drosophila disk large/ZO-1 (PDZ)-containing scaffolding protein that regulates a variety of physiological functions^11^,^12^, such as controlling the localization, delivery, surface stability and function of transporters, integral membrane proteins, and ion channel proteins, as well as clustering of proteins to specific cellular domains to facilitate signaling^13^,^14^.

Recently, EBP50 has been reported to have the capacity to bind to iNOS through one of its PDZ domains and control the localization of iNOS to model and mycobacterial phagosomes in macrophages^15^,^16^, enable macrophages to produce bactericidal nitric oxide (NO) at the vicinity of microbe-containing phagosomes, thereby promoting the elimination of intracellular microbes. However, some bacteria^16^,^17^, including *Mycobacterium bovis* BCG, have been reported to interfere with EBP50 and prevent the recruitment of iNOS to phagosomes, thus evading the direct damage from NO, the product of iNOS. Nevertheless, although *M. bovis* BCG has the capability to exclude iNOS by targeting EBP50, silencing the expression of EBP50 in macrophages still significantly increased the intracellular survival of mycobacteria^16^, which indicated that EBP50 may have some unknown antimycobacterial properties besides manipulating the distribution of iNOS. In this study, we investigated the effects and mechanisms of EBP50 overexpression on the colocalization of iNOS and *M. tuberculosis* and elimination of intracellular *M. tuberculosis* in RAW264.7 cells.

## Materials and Methods

### Materials

Caspase inhibitor carbobenzoxy-valylalanyl-aspartyl-[O-methyl]-fluoromethylketone (Z-VAD-FMK) were purchased from Sigma-Aldrich (St. Louis, MO). FuGene6 transfect regent was purchased from Promega Corporation (Promega, Madison, WI). iNOS inhibitor N-[3-(Aminomethyl)benzyl] acetamidine (1400 W), NO donor (Sodium nitroprusside, SNP), NO scavenger (Carboxy-PTIO) and caspase-3 activity kit were obtained from Beyotime Institute of Biotechnology (Haimen, Jiangsu, China). Antibodies specific for EBP50 and iNOS were obtained from Abcam (San Francisco, CA), and antibody specific for caspase-3 was obtained from Santa Cruz Biotechnology (Santa Cruz, CA). The lentiviral expression plasmid pLenti-146-GFP was kindly gifted by Dr. Senlin Li from the Department of Pharmacology, University of Texas Health Science Center, San Antonio. Plasmid pLenti-146 was constructed in our lab by deleting the GFP encoding fragment from pLenti-146-GFP. *M. tuberculosis*-GFP was constructed by transforming plasmid pUV15 (a kind gift from professor Michael Niederweis from the Department of Microbiology, University of Alabama, Birmingham) into *M. tuberculosis* H37Rv (ATCC 27294), and the resultant bacteria were screened on Middlebrook 7H10 agar (BD Company, Franklin Lakes, NJ) containing 50 μg/ml hygromycin B, as described previously^18^.

### Cell culture

RAW264.7 cells (ATCC, TIB-202) were cultured in Dulbecco’s modified Eagle medium (DMEM, Sigma-Aldrich) supplemented with 10% fetal bovine serum (FBS) and 1% penicillin-streptomycin. 293T cells (ATCC, TIB-202) were cultured in RPMI 1640 medium (GIBCO, Grand Island, NY) supplemented with 10% FBS and 1% penicillin-streptomycin (GIBCO). Cells were cultured in a standard tissue culture incubator at 37 °C with an atmosphere of 5% CO_2_ and 95% air.

### Bacterial culture

*M. smegmatis* mc^2^-155 (ATCC 700084), *M. tuberculosis* H37Rv (ATCC 27294), and *M. tuberculosis*-GFP were grown to early mid-log phase in Middlebrook 7H9 broth with or without hygromycin B, supplemented with 10% ADC and 0.5% glycerol at 37 °C. The bacterial aggregates were shattered by gentle agitation with 3-mm-diameter glass beads. The resultant bacteria were diluted in phosphate-buffered saline (PBS). The solution mixture was left standing for 15 min before the supernatant was collected and adjusted to an OD600 of 0.5 (about 10^7^ individualized bacteria/ml). The homogenate of bacteria was used to infect cells at the indicated multiplicity of infection (MOI).

### Lentivirus vector construction

The EBP50 encoding gene was amplified from RAW264.7 cells by reverse-transcription-polymerase chain reaction (RT-PCR). The primers containing *Age* I and *Sac* II sites for PCR were as follows, forward primer: 5′ATAACCGGTATGAGCGCGGACG3′; reverse primer: 5′AGGCCGCGGTCAGAGGTTGCTGAAGAGT 3′. The thermal cycling condition for PCR was 95 °C for 5 min, 30 cycles at 94 °C for 40 s, 55 °C for 30 s and 72 °C for 90 s, followed by a final round of 72 °C for 10 min. The PCR product was digested by *Age* I and *Sac* II, and ligated with linearized pLenti-146-GFP by T4 DNA ligase to form recombinant lentiviral expression vector pLenti-146-EBP50.

The siRNA sequence targeting EBP50 gene was designed using online siRNA software. The sequence of the shRNA cassette against EBP50 (shEBP50) is 5′-TGC AAT GGA GAG ATA CAG AAT TCA AGA GAT TCT GTA TCT CTC CAT TGC TTT TTT C-3′. The negative control shRNA sequence (shCon) is 5′-TGC TAA GCA CAG TAA GTG TAT TCA AGA GAT ACA CTT ACT GTG CTT AGC TTT TTT C-3′. The shRNA expressing vectors were designed by inserting annealed oligo sequences into the digested pSicoR vectors on the basis of the shRNA sequences described above, named pSicoR-EBP50 or pSicoR-Con.

### Lentiviral vectors package

293T cells were cultured into 6-well plates at 37 °C with 5% CO_2_ in humidified atmosphere. After reaching 70% ~ 80% confluence, 293T cells were triple transfected with the lentiviral expression vector pLenti-146-EBP50 and two packaging vectors pSPAX2 and pMD2.G as a group named LV-EBP50, and pLenti-146-GFP, pSPAX2 with pMD2.G as a group named LV-Lenti-GFP, pLenti-146, pSPAX2 with pMD2.G as a group named LV-Lenti, pSicoR-EBP50, pSPAX2 with pMD2.G as a group named LV-shEBP50 and pSicoR-Con, pSPAX2 with pMD2.G as a group named LV-shCon by using FuGene6 transfect regent following the manufacturers protocol. Culture supernatants were collected every 24 h for 3 days, filtered through a 0.45-μm pore size filter, and concentrated two times with ultracentrifugation at 50,000 × *g* for 120 min. Each group was diluted 1:10, 1:100, and 1:1000 times to determine virus titers. The virus particles were resuspended in sterile PBS, and stored at –80 °C until use.

### Real-time PCR

Total cellular RNA was extracted from cells using TRIzol reagent (Invitrogen, Carlsbad, CA), following the manufacturer’s instruction, then reverse-transcribed into complementary DNA using the PrimeScript RT reagent kit (Takara, Shiga, Japan) according to the manufacturer’s instructions. The expression of EBP50 was determined by real-time PCR using SYBR Premix Ex Taq (Takara, Shiga, Japan) and StepOnePlus Real-Time PCR System (Applied Biosystems, CA). Primers used for PCR amplification were: GAPDH: 5′-GCACCGTCAAGGCTGAGAAC-3′ (forward), 5′-TGGTGAAGACGCCAGTGGA-3′ (reverse); EBP50: 5′-CCAGGACCGAATTGTGGA-3′ (forward), 5′- CCTGGGATGGGATCACTTTG -3′ (reverse). The mRNA levels of EBP50 relative to the control was calculated by 2^−△△^CT.

### Western blotting

The cultured cells were collected and lysed using RIPA lysis buffer containing 1 mM phenylmethylsulfonyl fluoride and 1% (vol/vol) protease inhibitor cocktail (Beyotime, Haimen, Jiangsu, China). Lysates were centrifuged at 12,000 × *g* for 15 min, and the protein concentration was measured using a bicinchoninic acid (BCA) Protein Assay kit (Pierce Biotechnology, Inc., Rockford, IL). Equal amounts of proteins were separated on sodium dodecyl sulfate polyacrylamide gel electrophoresis (SDS-PAGE) gels and transferred to polyvinylidene fluoride membranes at 4 °C for 1 h. Membranes were blocked in 5% BSA and incubated with primary antibodies against EBP50, iNOS and caspase-3 at 4 °C overnight. Then membranes were incubated at room temperature for 1 h with relevant secondary antibodies, and blots were visualized using enhanced chemiluminescence (ECL; Thermo Pierce, Rockford, Illinois, USA) according to the manufacturer’s instructions and quantified by densitometry using Quantity One image software with β-actin used as an internal control.

### Phagocytosis assay by flow cytometry (FCM)

RAW264.7 cells transduced with or without recombinant lentivirus were challenged by *M. tuberculosis*-GFP at the MOI of 10. After 4 h incubation, cells were washed thoroughly with D-Hanks buffer to remove extracellular bacteria. Then cells were collected and analyzed using Cytomics FC 500 flow cytometer (Beckman Coulter Inc., Brea, CA, USA).

### Nitric oxide production detection

The levels of NO were measured by commercial kits (Jiancheng Bioengineering Institute, Nanjing) according to the manufacturers’ protocols. Briefly, RAW264.7 cells were transduced with or without recombinant lentivirus for 72 h, the supernatants were collected for detection. Griess reagent (50 μL; equal volume of 1% sulfanilamide in HCl 0.1 mol/L and 0.1% N-[(-1-naphthyl)-ethylenediamine dihydrochloride] was added to 50 μL of supernatants. Nitrite concentration was determined by spectrophotometry (540 nm) from a standard curve (0-100 mmol/L) derived from NaNO_2_. NO data was expressed as mean ± SD (nitrite) in μmol/L.

### Confocal microscopy

RAW264.7 cells were cultured on collagen-precoated glass coverslips in 24-well plates, transduced with or without recombinant lentivirus for 48 h, then infected with *M. tuberculosis*-GFP at an MOI of 5. At 4 h after the infection, cells were washed thoroughly with D-Hanks buffer to remove extracellular bacteria and cultured in DMEM medium containing 10% FBS for 24 h. Cells were fixed with 4% paraformaldehyde followed by membrane permeabilization using 0.2% Triton X-100, blocked with 5% BSA and incubated with primary and then fluorophore-conjugated secondary antibodies before mounting. The colocalization of iNOS and *M. tuberculosis* was viewed by confocal laser scanning microscopy (Zeiss Axiovert, LSM710). Twenty high-power fields were randomly selected to count the colocalization of iNOS and *M. tuberculosis*-GFP. The observer was blinded to the treatment of each sample.

### Colony-forming units

RAW264.7 cells were transduced with or without recombinant lentivirus for 72 h, in the presence or absence of 20 μM Z-VAD-FMK, and then challenged with H37Rv at an MOI of 10. After 4-h incubation, cells were washed thoroughly with cold PBS to remove extracellular bacteria. Then infected cells were incubated for the indicated time. Cells were lysed with 0.01% SDS, and serial 10-fold dilutions of each sample were plated on Middlebrook 7H10 agar plates. Colonies were counted after four-week incubation.

### Apoptosis analysis by flow cytometry

RAW264.7 cells were incubated in six-well plates in the absence or presence of SNP, 1400W or Carboxy-PTIO for 1 h, followed by transduction with LV-EBP50 or LV-Lenti for 72 h, then infected with *M. tuberculosis* H37Rv at the MOI of 5 for 24 h. The apoptotic ratios of RAW264.7, RAW264.7/LV-EBP50, RAW264.7/LV-Lenti, RAW264.7/SNP/LV-Lenti, RAW264.7/1400W/LV-EBP50, RAW264.7/Carboxy-PTIO/LV-EBP50, RAW264.7/H37Rv, RAW264.7/LV-EBP50/H37Rv, RAW264.7/LV-Lenti/H37Rv cells were measured by FCM with Annexin V-PE kit (ebioscience, San Diego, CA, USA).

### Caspase-3 activity assay

Activity of caspase-3 was measured colorimetrically using the caspase-3 assay kit, which is based on the ability of caspase-3 to convert acetyl-Asp- Glu-Val-Asp p-nitroanilide into p-nitroaniline. Caspase-3 activity was quantified in the samples with a microplate spectrophotometer at an absorbance of 405 nm. Caspase-3 activity was qualified as fold enzyme activity compared with that of the untreated RAW264.7 cells.

### Statistical analysis

All the presented data and results were confirmed in at least three independent experiments. Unpaired Student’s t test or one-way analysis of variance was used to determine the significance of the results from real-time RT-PCR experiments and colony-forming unit assays, the colocalization of *M. tuberculosis* with iNOS. Data were considered statistically significant at *P* < 0.05.

## Results

### Overexpression of EBP50 did not enhance the iNOS recruitment to M. tuberculosis phagosomes

**Figure 1:**
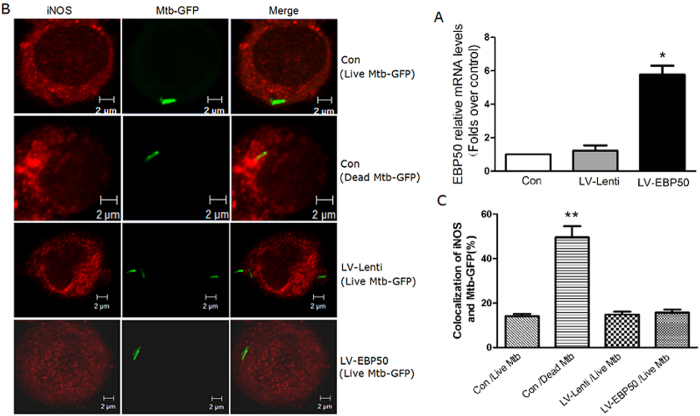
EBP50 has no significant impact on the recruitment of iNOS to live *M. tuberculosis*-containing phagosomes. RAW264.7 cells were left untreated (Con) or transduced with LV-EBP50 or LV-Lenti for 72 h, followed by infection with live or heat-killed *M. tuberculosis*-GFP (Mtb-GFP) at the MOI of 10. The mRNA expression levels of EBP50 were measured by real-time PCR at 72h after the transduction (**A**) . After 4-h chase of *M. tuberculosis*, cells were fixed and stained for iNOS. The localization of iNOS (red) and Mtb-GFP (green) were examined by confocal microscopy **(B)**. Twenty high-power fields were randomly selected and the colocalization of iNOS and Mtb-GFP was counted in each condition **(C)**. Data are represented as mean ± SEM of at least three independent experiments. *P < 0.05, **P < 0.01 compared with the untreated group.

EBP50 has been reported to control the distribution of iNOS in macrophages, promote the recruitment of iNOS to phagosomes containing latex beads and dead BCG, thereby enabling the macrophages generating NO approaching the bacteria-containing phagosome^15^,^16^. However, live BCG can inhibit the recruitment of iNOS to phagosomes^16^. To explore whether EBP50 can affect the distribution of iNOS on phagosomes containing live *M. tuberculosis*, RAW264.7 cells were transduced with LV-EBP50 and LV-Lenti at the MOIs of 10 for 72 h, then infected with *M. tuberculosis*-GFP and analyzed by laser scan confocal microscope for localization of endogenous iNOS to *M. tuberculosis*-GFP. Data showed no significant increase in iNOS recruitment to *M. tuberculosis*-GFP in LV-EBP50-transduced macrophages compared with untreated cells (15.7% ± 5.3% vs. 14.1% ± 3.6%, *P* > 0.05) and LV-Lenti transduced cells (15.7% ± 5.3% vs. 14.8% ± 4.9%, *P* > 0.05) at 4 h after infection (Fig. 1). Analogous results were also observed at 12 h and 24 h post infection (data not shown). This indicated that EBP50 overexpression has no significant effect on the recruitment of iNOS to *M. tuberculosis*-containing phagosomes.

### EBP50 enhanced the intracellular killing but not phagocytosis of M. tuberculosis in macrophage

**Figure 2:**
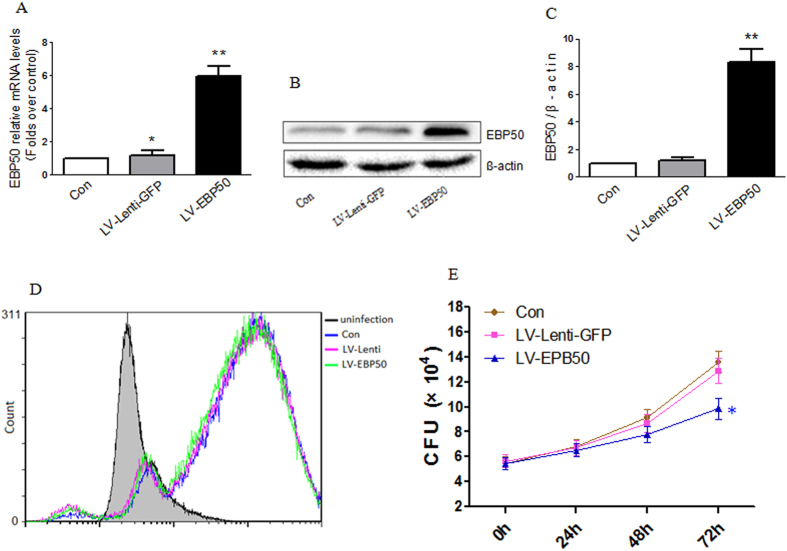
Overexpression of EBP50 promotes the killing of intracellular *M. tuberculosis* in RAW264.7 cells. RAW264.7 cells were left untreated (Con) or transduced with LV-EBP50 or control lentivirus (LV-Lenti-GFP) for 72 h. The mRNA expression levels of EBP50 were measured by real-time PCR **(A)**. The protein levels were detected by Western blotting **(B)**. Band density of the specific protein was analyzed with Quantity One image software, and the results were expressed as average density to β-actin **(C)**. RAW264.7 cells were left untreated (Con) or transduced with LV-EBP50 or LV-Lenti for 72 h, followed by infection with *M. tuberculosis*-GFP or *M. tuberculosis* H37Rv at the MOIs of 10. Phagocytosis of *M. tuberculosis*-GFP was detected by flow cytometry at 4 h postinfection **(D)** and intracellular mycobacteria survival was detected by CFU counting at the indicated time points postinfection **(E)**. Data are represented as mean ± SEM of at least three independent experiments. *P < 0.05, **P < 0.01 compared with the uninfected group.

To explore whether EBP50 plays a role in phagocytosis and killing of *M. tuberculosis*, LV-EBP50 and control lentivirus were used to transduce RAW264.7 cells. The expressions of EBP50 were determined by real-time PCR and Western blotting after 72-h infection of viral particles. Results showed that both the mRNA levels and the protein expressions of EBP50 were significantly increased after LV-EBP50 transduction (Fig. 2A–C). Then the effects of EBP50 on the phagocytosis and killing of *M. tuberculosis* were examined by flow cytometry and colony forming unit (CFU) assay, respectively. Data showed that the increase of EBP50 expression did not affect the phagocytosis capacity of RAW264.7 cells to *M. tuberculosis* (Fig. 2D). However, the intracellular survival of *M. tuberculosis* in RAW264.7 cells was significantly decreased after the transduction of LV-EBP50 (Fig. 2E), which indicated that EBP50 can promote the elimination of *M. tuberculosis* in macrophages.

### Overexpression of EBP50 increased the expression of iNOS and generation of NO in macrophages

**Figure 3:**
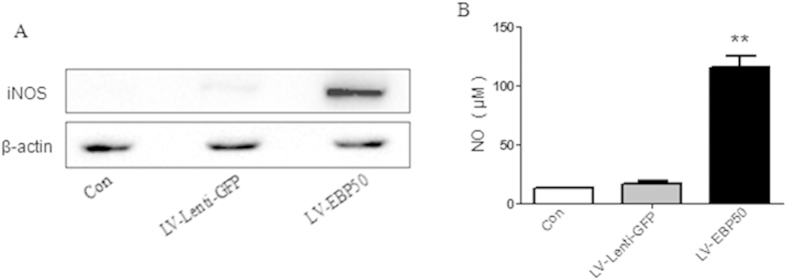
Overexpression of EBP50 increased the expression of iNOS and generation of NO. RAW264.7 cells were left untreated (Con) or transduced with LV-EBP50 or LV-Lenti-GFP for 72 h. The amount of iNOS was detected by Western blotting. β-actin was used as a loading reference **(A)**. The production of NO was measured by Griess reagent **(B)**. Data are represented as mean ± SEM of three independent experiments. **P < 0.01 compared with the untreated group.

In the assay of confocal laser scanning microscopy, the result of immunostaining with labeled anti-iNOS antibody showed that the expression of iNOS was seemingly higher in LV-EBP50 transduced macrophages when compared with LV-Lenti transduced and untreated cells. To affirm the expression level of iNOS in the condition of EBP50 overexpression, RAW264.7 cells were transduced with LV-EBP50 and LV-Lenti-GFP for 72 h, then the expression of iNOS was detected by Western blotting, and the level of NO was measured by Griess reagent method. Results showed that the expression of iNOS was significantly upregulated upon overexpression of EBP50 (Fig. 3A). As expected, EBP50 significantly increased NO production in RAW264.7 cells (Fig. 3B).

### EBP50 promoted elimination of intracellular M. tuberculosis by inducing apoptosis in macrophages

**Figure 4:**
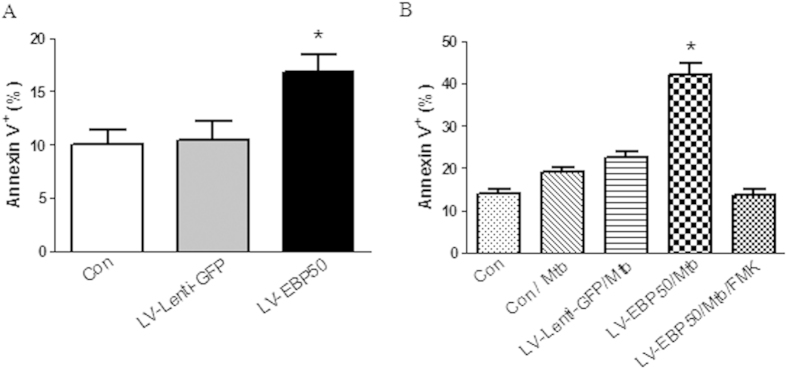
EBP50 promotes the apoptosis in macrophages. RAW264.7 cells were left untreated (Con) or transduced with LV-EBP50 or LV-Lenti-GFP for 72 h. The apoptosis ratio was measured by FCM **(A)**. RAW264.7 cells were left untreated or treated with Z-VAD-FMK at a concentration of 200 μM along with LV-EBP50 or LV-Lenti-GFP transduction, followed by infection with *M. tuberculosis* (Mtb), then analyzed for apoptosis ratio by FCM **(B)**. Data are represented as mean ± SEM of at least three independent experiments. *P < 0.05 compared with untreated group.

RAW264.7 cells were left untreated or transduced with LV-EBP50 or LV-Lenti-GFP for 72 h, then analyzed for apoptosis by FCM. Data demonstrated that EBP50 overexpression distinctly enhanced the apoptotic ratios of cells compared with untreated RAW264.7 cells (16.89% ± 2.0% vs. 10.04% ± 1.7%, *P* < 0.05) (Fig. 4A).

To exam whether EBP50 can synergize with *M. tuberculosis* infection in inducing macrophage apoptosis, RAW264.7 were transduced with LV-EBP50 and LV-Lenti-GFP at the MOIs of 10 for 72 h, then infected with *M. tuberculosis* for 24 h and analyzed by FCM for cell apoptosis. Results showed that EBP50 overexpression distinctly increased the *M. tuberculosis* infection-induced apoptosis of RAW264.7 cells when compared with LV-Lenti-GFP-transduced RAW264.7 cells (19.19% ± 2.4% vs. 42.20% ± 3.2%, *P* < 0.05) (Fig. 4B).

**Figure 5:**
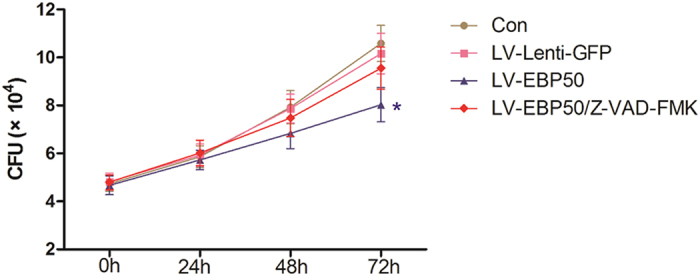
Inhibition of apoptosis abrogates the EBP50-induced increase of bactericidal capability in macrophage. RAW264.7 cells were treated with Z-VAD-FMK and transduced with LV-Lenti-GFP or LV-EBP50, followed by infection with *M. tuberculosis*. The intracellular mycobacteria survival was determined by CFU counting at the indicated time points. Data are represented as mean ± SEM of at least three independent experiments. *P < 0.05 compared with untreated group.

Since apoptosis has been demonstrated to play an important role for macrophages in defense against intracellular pathogens^1^,^19^, we hypothesized that the enhancement of bactericidal capability in LV-EBP50-transduced macrophages may be because of the increase of apoptosis induced by EBP50. To test this hypothesis, Z-VAD-FMK, a broad-spectrum caspase inhibitor was used to inhibit the apoptosis of RAW264.7 cells along with LV-EBP50 transduction. The macrophages were then infected with *M. tuberculosis* at the MOIs of 10, and the intracellular mycobacteria survival was detected by counting the CFUs. Data showed that Z-VAD-FMK significantly decreased the apoptosis ratio of RAW264.7 cells (Fig. 4B), and abrogated the EBP50-induced increase of bactericidal capability in macrophage (Fig. 5), which suggested that EBP50 promoted elimination of intracellular *M. tuberculosis* by inducing apoptosis in macrophages.

### Apoptosis induced by EBP50 was nitric oxide dependent

**Figure 6:**
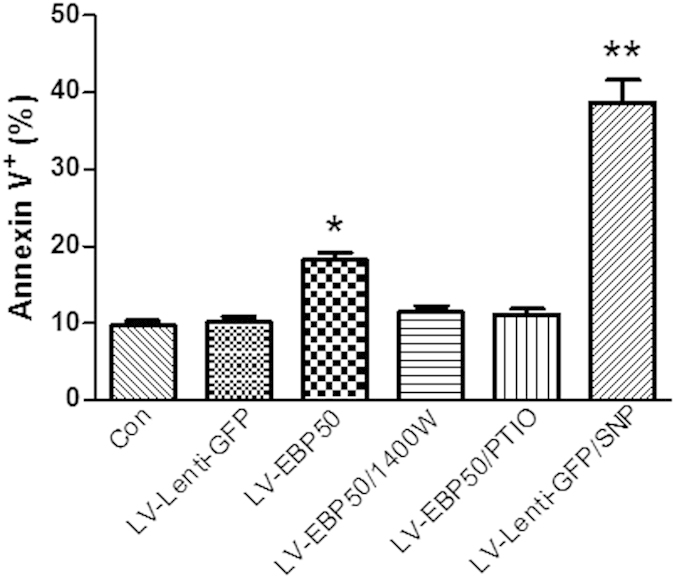
Apoptosis induced by EBP50 was nitric oxide dependent. RAW264.7 cells were left untreated (Con) or treated with NO donor (SNP), iNOS inhibitor (1400W) or NO scavenger (Carboxy-PTIO) for 1 h, followed by transduction with LV-EBP50 or LV-Lenti-GFP for 72 h. The apoptosis incidence was measured by FCM. Data are represented as mean ± SEM of at least three independent experiments. *P < 0.05, **P < 0.01 compared with untreated group.

Aforementioned data demonstrated that EBP50 promotes the generation of NO and increases the apoptosis ratio in macrophages. NO is a bactericidal reactive-free radical, and has been reported to be involved in the regulation of cell death^20^,^21^,^22^. To test whether NO plays a role in the apoptosis of macrophages induced by EBP50, RAW264.7 cells were left untreated or treated with NO donor (SNP) at a concentration of 200 μM, iNOS inhibitor (1400W) at a concentration of 20 μM, or NO scavenger (Carboxy-PTIO) at a concentration of 200 μM for 1 h, followed by transduction with LV-EBP50 or LV-Lenti-GFP for 72 h, and apoptosis incidence was measured by FCM with commercial Annexin V-PE kit. Results showed that both NO inhibitor and NO scavenger treatment led to significant decrease of apoptosis of LV-EBP50-transduced RAW264.7 cells, while NO donor significantly increased the apoptosis of LV-Lenti-GFP-transduced cells (Fig. 6).

### EBP50 induces apoptosis by increasing the expression of Bax and caspase-3

**Figure 7:**
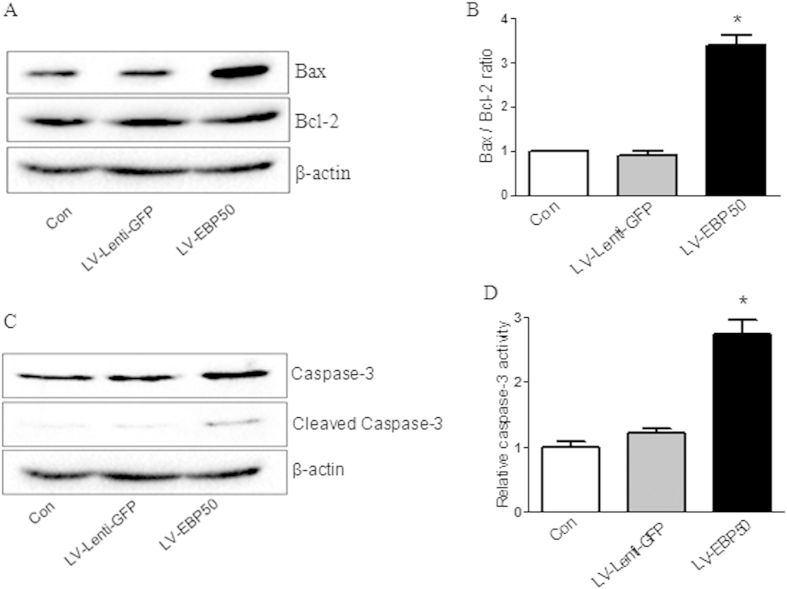
EBP50 induces apoptosis by increasing the expression of Bax and caspase-3. RAW264.7 cells were left untreated (Con) or transduced with LV-EBP50 or LV-Lenti-GFP for 72 h. The expression levels of Bcl-2, Bax **(A)** and caspase-3 **(C)** were determined by Western blotting. Band density of the specific protein was analyzed with Quantity One image software and the Bax/Bcl-2 ratio was quantified **(B)**. The activity of caspase-3 was measured colorimetrically using the caspase-3 assay kit **(D)**. Data are represented as mean ± SEM of at least three independent experiments. *P < 0.05 compared with the untreated group.

NO was previously reported to induce apoptosis by enhancing Bax-to-Bcl-2 ratio and activating caspase-3^23^. EBP50 was also reported to promote the apoptosis of pancreatic cancer cells by decreasing the expression levels of Bcl-2^24^. To explore the molecular mechanisms underlying the EBP50-induced apoptosis in macrophages, RAW264.7 cells were left untreated or transduced with LV-EBP50 or LV-Lenti-GFP at the MOIs of 10 for 72 h, then the expression levels of Bcl-2, Bax, and caspase-3 were determined by Western blotting. The activities of caspase-3 in RAW264.7 cells were also measured. Results showed that the overexpression of EBP50 significantly increased the expression of Bax and caspase-3, the activity of caspase-3 was also increased upon EBP50 overexpression, while the expression of Bcl-2 had no distinct change (Fig. 7), which suggested that the proapoptotic effect of EBP50 overexpression occurred due to the increase of Bax expression and activation of caspase-3.

### *M. tuberculosis* infection downregulated the expression of EBP50

**Figure 8:**
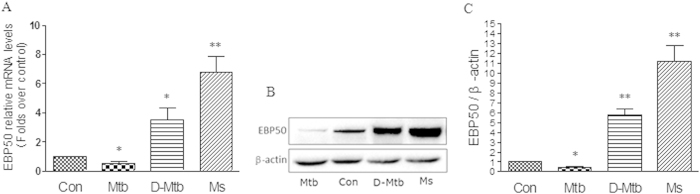
EBP50 expression in RAW264.7 cells after mycobacteria infection. RAW264.7 cells were left uninfected (Con) or infected with *M. tuberculosis* H37Rv (Mtb), heat-killed *M.tuberculosis* (D-Mtb) or *M. smegmatis* mc2-155 (Ms) at the MOIs of 10 for 24 h. mRNA expression levels of EBP50 were measured by real-time PCR **(A)**. EBP50 protein levels were measured by Western blotting. β-actin was used as a loading reference **(B)**. Band density of the specific protein was analyzed with Quantity One image software, and the results were expressed as average density to β-actin **(C)**. Data are represented as mean ± SEM of at least three independent experiments. *P < 0.05, **P < 0.01 compared with uninfected group.

**Figure 9:**
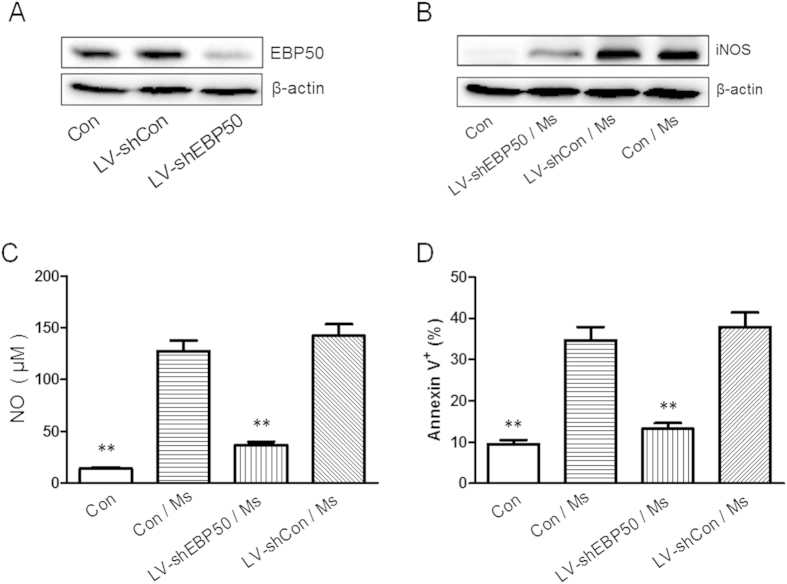
EBP50 knockdown decreases the levels of iNOS, NO and apoptosis ratios of RAW264.7 cells following mycobacteria infection. RAW264.7 cells were left untreated (Con) or transduced with LV-shEBP50 or LV-shCon for 72 h. The expression levels of EBP50 were determined by Western blotting **(A)**. RAW264.7 cells were left untreated (Con) or transduced with LV-shEBP50 or LV-shCon for 72 h, then infected with *M. smegmatis* mc2-155 (Ms) at the MOIs of 10 for 24 h. The amount of iNOS was detected by Western blotting. β-actin was used as a loading reference **(B)**. The production of NO was measured by Griess reagent **(C)** and the apoptosis incidence was measured by FCM **(D)**. Data are represented as mean ± SEM of at least three independent experiments. **P < 0.01 compared with LV-shEBP50 transduction group.

It is well known that when compared to avirulent mycobacteria, virulent mycobacteria can inhibit the expression of iNOS and production of NO, decrease the apoptosis level of macrophage. To test whether this difference correlates with the expression of EBP50, RAW264.7 cells were challenged with *M. tuberculosis* H37Rv, heat-killed *M. tuberculosis* H37Rv and *M. smegmatis* mc2-155, a fast-growing non-pathogenic mycobacteria, and then analyzed for EBP50 expression by real-time PCR and Western-blotting. Data showed that the mRNA level of EBP50 was significantly upregulated after *M. smegmatis* and dead *M. tuberculosis* infection, while the mRNA levels of EBP50 was significantly downregulated after *M. tuberculosis* infection (Fig. 8A). The results of Western blot analysis showed that the protein expressions of EBP50 were also upregulated by *M. smegmatis* and dead *M. tuberculosis*, downregulated by *M. tuberculosis* (Fig. 8B). Next, lentiviral vector-based shRNA targeting EBP50 gene was constructed and transduced into RAW264.7 cells, followed by the infection with *M.smegmatis*. Results showed that the expression of EBP50 was significantly downregulated following the transduction of EBP50-targeting shRNA encoding lentivirus (Fig. 9A). Furthermore, EBP50-targeting shRNA encoding lentivirus significantly reduced the expression of iNOS, the production of NO and the apoptosis ratios of RAW264.7 cells following the infection of *M.smegmatis* (Fig. 9B–D).

## Discussion

EBP50 is a scaffolding protein that allows sorting and localization of cell proteins to specific cellular domains by binding to the cytoskeleton through its two PDZ domains^11^,^12^,^13^,^14^. In macrophages, EBP50 was reported to play a key role in controlling the recruitment of iNOS to phagosome^15^,^16^. NO is a highly reactive and diffusible free radical with antimycobacterial properties that is generated by iNOS^20^,^25^. The proximity of iNOS to the bacteria-containing phagosome thus facilitates macrophage to generate NO in the vicinity of its phagosome and clear infected bacteria^26^. Nevertheless, It was reported that *M. bovis* BCG, an attenuated vaccine strain of *M. bovis* inhibits trafficking of iNOS to the live BCG-containing phagosome by releasing EBP50 from phagosomal membrane^16^, which indicated that live mycobacteria may have the capability to disrupt the function of EBP50.

*M. tuberculosis* is the pathogen that causes tuberculosis and has the capability to survive and persist in macrophages. It has evolved mechanisms to counter multiple and often independent bactericidal effectors in macrophages^27^,^28^,^29^,^30^. However, whether *M. tuberculosis* can prevent the recruitment of iNOS to phagosomes in macrophages by disrupting the function of EBP50, like *M. bovis* BCG, remains unclear. Furthermore, although *M. bovis* BCG can inhibit the recruitment of iNOS to phagosomes in macrophages, knockdown of endogenous EBP50 still promoted the intracellular survival of mycobacteria^16^, which demonstrate that EBP50 may have some unknown antimycobacterial properties besides manipulating the distribution of iNOS.

In this study, we transduced RAW264.7 cells with a recombinant lentivirus encoding EBP50 gene. Results showed that there was no significant increase in iNOS recruitment to *M. tuberculosis* in LV-EBP50-transduced macrophages compared with untreated cells and LV-Lenti-GFP transduced cells. Although we did not perform the dynamic monitoring of iNOS to *M. tuberculosis*-containing phagosomes due to the limitation of equipment, our results suggested that *M. tuberculosis* is able to prevent the recruitment of iNOS to *M. tuberculosis*-containing phagosomes. However, results showed that overexpression of EBP50 in RAW264.7 cells significantly increased the intracellular killing of *M. tuberculosis*. These data indicated that EBP50 can promote the intracellular killing of *M. tuberculosis* in macrophages through some mechanisms other than controlling of iNOS distribution.

In the assay of confocal laser scanning microscopy, we found that the expression of iNOS was seemingly higher in LV-EBP50-transduced macrophages. The upregulation of iNOS upon the overexpression of EBP50 was subsequently confirmed by Western blot analysis and NO determination. Thus this study provides the first evidence, to the best of our knowledge, that EBP50 overexpression may promote the expression of iNOS and generation of NO in macrophages.

Several studies have shown that besides its function as a reactive free radical to execute direct antimicrobial activities, NO plays a role in innate immunity by inducing macrophage apoptosis^21^,^31^. In this study we also found that EBP50 overexpression significantly increased the apoptosis ratio in RAW264.7 cells. Apoptosis has been demonstrated to play a role for macrophages in defense against intracellular pathogens. But there are some controversies with regard to the roles of NO-induced apoptosis in macrophages for defending against mycobacteria^32^. To explore the roles of EBP50-induced apoptosis in the bactericidal effect of macrophage on intracellular *M. tuberculosis*, RAW264.7 cells transduced with LV-EBP50 were left untreated or treated with apoptosis inhibitor, and then analyzed for the apoptosis ratio and antimycobacterial effect. Data showed that inhibiting apoptosis abrogated the intracellular killing of *M. tuberculosis* in LV-EBP50-transduced macrophages, which indicated that EBP50 promoted the elimination of intracellular *M. tuberculosis* by inducing apoptosis in macrophages. Activating the programmed death of host cells is one of the important protective mechanisms for host to defend against the infection of intracellular pathogens. Numerous studies have shown that avirulent mycobacteria induce apoptosis in macrophages while virulent strains do not or even inhibit apoptosis in macrophage, which indicated that disrupting the activation of apoptosis in macrophages is a virulence mechanism for *M. tuberculosis* to achieve immune evasion^33^,^34^. Protective immune mechanism for macrophage involves the role of EBP50 in inducing apoptosis.

We further explored the underlying molecular mechanisms of apoptosis upregulation upon EBP50 overexpression. Results showed that the increase of apoptosis levels in LV-EBP50-transduced macrophages can be abrogated by iNOS inhibitors or NO scavengers. On the contrary, NO donor can promote the apoptosis ratios of control lentivirus-transduced macrophages. Furthermore, EBP50 overexpression obviously increased the expression of pro-apoptosis protein Bax and caspase-3. These data indicated that the apoptosis induced by EBP50 was NO dependent and mediated by Bax and caspase-3.

Overall, this study demonstrated for the first time, to the best of our knowledge, that EBP50 upregulates the apoptosis ratio of macrophage by increasing the expression of Bax and caspase-3 in an NO-dependent mechanism, and promotes the intracellular killing of *M. tuberculosis*. This might be one of the protective mechanisms of host macrophages to clear infected *M. tuberculosis*. In this study, we found that *M. tuberculosis* has the capability to suppress the expression of EBP50 in macrophages, while the infection of *M. smegmatis*, a non-pathogenic mycobacterium, obviously increased the expression of EBP50. This phenomenon is consistent with the fact that when compared with *M.tuberculosis,* the infection of avirulent mycobacteria, including *M. smegmatis*, dramatically increases the expression of iNOS, the production of NO and the apoptosis level of host macrophages^35^,^36^,^37^,^38^. Thus, the expression level of EBP50 in macrophages might be affected by the virulence of infected mycobacteria. Suppressing the expression of EBP50 in macrophages might be one of the immune-evading mechanisms of *M. tuberculosis*.

## Additional Information

**How to cite this article**: Guo, Y. *et al.* EBP50 induces apoptosis in macrophages by upregulating nitric oxide production to eliminate intracellular *Mycobacterium tuberculosis. Sci. Rep.* **6**, 18961; doi: 10.1038/srep18961 (2016).
